# Group singing in bereavement: effects on mental health, self-efficacy, self-esteem and well-being

**DOI:** 10.1136/bmjspcare-2018-001642

**Published:** 2019-06-26

**Authors:** Daisy Fancourt, Saoirse Finn, Katey Warran, Theresa Wiseman

**Affiliations:** 1 Department of Behavioural Science and Health, University College London, London, UK; 2 Centre for Performance Science, Royal College of Music, London, UK; 3 Applied Health Research, The Royal Marsden NHS Foundation Trust, London, UK

**Keywords:** bereavement, psychological care, depression

## Abstract

**Objectives:**

Bereavement is associated with negative affective, cognitive, behavioural and physiological responses. However, factors, such as coping, self-efficacy and self-esteem, can buffer negative effects of grief, and can be increased through mutual support interventions, such as shared leisure activities. This study used a non-randomised controlled design to explore the effects of group choir singing on mental health among people who have been bereaved due to cancer.

**Methods:**

A total of 58 adults bereaved in the last 5 years who had not started psychological therapy in the last 12 weeks or medication for anxiety or depression in the last month were recruited and elected to join a choir (n=29) or participate in the non-intervention control group (n=29). Joining a choir involved engaging in 90 min weekly singing and social sessions for 12 weeks with a post-intervention assessment at week 24. We used linear mixed effects models adjusted for demographics, health-related variables, musical engagement and time since bereavement to model changes over time between the two groups in symptoms of anxiety, depression, well-being, self-efficacy and self-esteem.

**Results:**

Participants who sang in a choir had more stable symptoms of depression and levels of well-being, as well as gradual improvements in their sense of self-efficacy and self-esteem over the 24 weeks. In contrast, those in the control group showed gradual increases in depressive symptoms, reductions in levels of well-being and self-esteem and no improvement in their self-efficacy. These results were independent of all covariates.

**Conclusions:**

Weekly group singing could be a promising mutual support intervention for people experiencing grief.

**Trial registration number:**

NCT02756780.

## Introduction

In the initial few years following bereavement, people report diverse psychological outcomes. Most common are affective responses (including depression, anxiety, guilt, loneliness and anger), cognitive responses (including denial, lowered self-esteem, helplessness and intrusive rumination), behavioural responses (including fatigue, agitation and social withdrawal) and physiological–somatic responses (including sleep disturbances, loss of appetite and exhaustion).^1^ Notably, recovery from these responses can be slow, with studies routinely finding persistent challenges several years after bereavement.^2 3^

However, these psychological responses vary according to a number of factors, including age, education, social support, previous experiences of grief, quality of relationship with the person lost, concurrent life stressors, financial situation, time since death and the cognitive interpretation of the loss.^1 4^ This last factor has gained interest as part of broader theoretical work on cognitive coping strategies,^5^ and is particularly appealing as it is potentially modifiable through different interventions. Cognitive interpretation research builds on work in social cognitive theory, which has highlighted the importance of self-evaluation (the determination of discrepancies between one’s current state and a desired outcome) and how success or failure in achieving a desired outcome provides the basis for judgements of an individual’s coping self-efficacy.^6^ Coping self-efficacy has been found to affect psychological and physical outcomes from a range of traumatic events from terrorist attacks to natural disasters, acting as a significant predictor of lower levels of grief over time, partly through its role as a focal mediator of recovery.^7 8^ Studies on bereavement self-efficacy have found associations with lower emotional distress and higher well-being and perceived physical health.^9^ Consequently, it has been proposed that people who have been bereaved could benefit from support to target self-efficacy.^9^

Research on the importance of coping self-efficacy also ties in with research suggesting that people who experience bereavement can, over the long-term, become more resilient. Personal growth, including increased self-understanding, maturity and ability to regulate affect, has been found among some people in response to grief.^10^ A key feature within this is whether an individual can move from rumination to more deliberate and constructive thoughts that eventually allow them to find meaning in the death experience and successfully rebuild functional assumptive world beliefs.^11^ Research suggests that self-esteem is an important factor within this, helping to reduce mental health problems in children and older adults facing bereavement.^12–15^ However, the challenge is how to support individuals in becoming more resilient, including enhancing their self-esteem.

There are a number of interventions and intervention models used to provide support in bereavement. While medical approaches, such as the use of antidepressants, can have a value in relieving symptoms of acute grief, there is controversy as to whether they interfere with adaptive processes involved in grieving and evidence does not suggest that they can support important targets, such as self-efficacy or self-esteem.^16^ Among other approaches, psychotherapeutic interventions, such as counselling, provide targeted individual support that can be tailored to individual circumstance, while mutual support approaches, such as support groups, provide members with opportunities to share coping techniques, reinforce positive change and normalise their situation. In particular, mutual support approaches can support individuals in ‘restoration-oriented coping’, which includes distracting oneself from grief, doing new things, and crafting new roles, identities and relationships; all associated with enhanced self-efficacy and self-esteem.^1^ Within mutual support approaches, shared social activities, such as leisure activities, in particular, have been highlighted as effective as they can help to induce positive emotions and support ‘compensation’, providing a substitute for a loss of identity and caring ‘role’ and deficits in social integration following bereavement.^17–19^

A shared social intervention receiving increasing research interest in relation to mental health, generally, is group singing. A large number of studies have demonstrated the psychological and emotional benefits of singing interventions in different populations, including older adults, women with postnatal depression, people who are homeless and people with long-term conditions.^20–22^ Specifically relating to bereavement, singing is practised in cultures globally as a way of providing support following loss.^23 24^ However, to date, there remain few empirical studies focusing on potential singing interventions in supporting those who have been bereaved. Group singing can provide distraction, new experiences and opportunities for new identities and relationships so could be a promising mutual support intervention. Consequently, this multisite longitudinal controlled study explored the impact of 6 months of weekly singing sessions on mental health, well-being, self-esteem and self-efficacy in people who had been bereaved.

## Methods

### Participants and procedure

**Figure 1:**
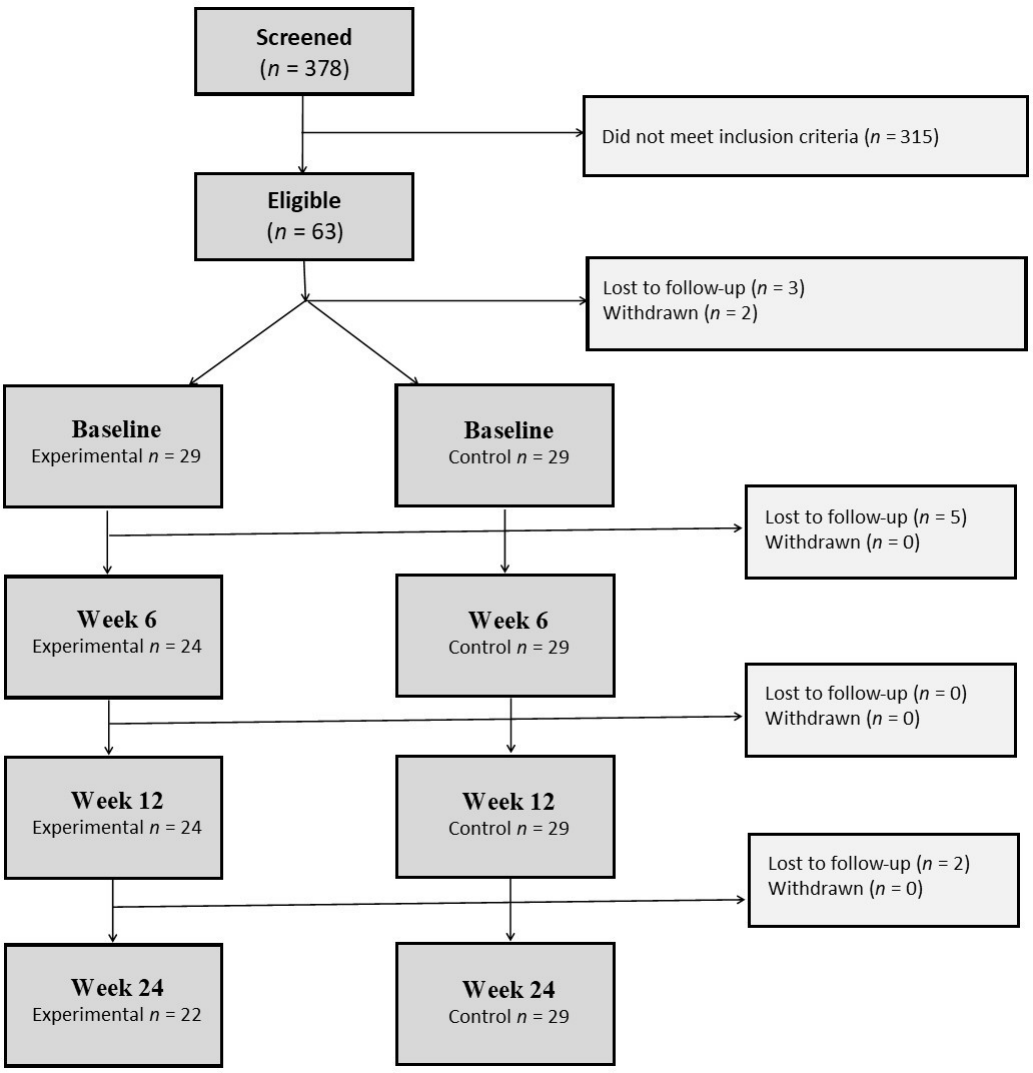
Consort diagram of participants participated in the study.

As research suggests that bereavement due to sudden and violent losses is associated with different patterns of mental health,^25^ we focused specifically on bereavement as a result of long-term illness, namely cancer. To be eligible, participants had to have lost a partner or close relative to cancer in the last 5 years. Participants were recruited by National Health Service hospital trusts across Greater London, and by the research team who visited support groups, hospital health days, and community and charity events. Social media was also widely used, as well as word of mouth from the choir. Participants were excluded if they were under the age of 18 years, if they were already engaged in a weekly choir, if they had started a formal course of psychological therapy in the last month or were scheduled to start one in the next 12 weeks, if they had started any new medication for anxiety or depression in the last month or if their level of English was insufficient to complete the questionnaires required. In the study, a total of 378 participants were screened for enrolment, 63 were eligible and 58 (29 control and 29 experimental) took part in the study. Of these, 51 completed the full 24 weeks and the other 7 were lost to follow-up (stopped attending sessions or providing data) (see figure 1). No incentives to participate in the study were offered to any participants.

As this was a longitudinal non-randomised controlled study, on enrolment, participants were given the option of joining a weekly choir for 12 weeks. Participants who did not select to join a choir formed the control group, while participants who did select to join a choir formed the experimental group. Joining a choir involved a weekly 90 min choir session comprising 60 min of singing and 30 min of socialising with tea and biscuits. The singing was led by a professional choir leader and consisted of 10 min of warm-up exercises, 30 min of learning new songs of contemporary popular repertoire and 20 min of singing songs familiar to the group from previous rehearsals. Two choirs were provided as study sites, run by Tenovus Cancer Care following the protocol described in previous studies.^26^ These two choirs were established for the purpose of this study and consisted of people affected by cancer, including patients, carers, those who had been bereaved and hospital staff. Following the initial 12-week intervention, participants were given the opportunity to continue with the choir activity if they chose. 12 participants (41%) chose to leave and the remaining 59% chose to continue attending. There were no statistical differences in any baseline measures between those who dropped out versus those who chose to continue with the choirs. All participants were asked to complete questionnaires at baseline, 6 and 12 weeks as well as at 3-month follow-up postintervention assessment (24 weeks).

### Measures

Our primary outcome measures were mental health and well-being. Symptoms of anxiety and depression were measured using the Hospital Anxiety and Depression Scale, ranging from 0 to 21 for each construct with higher scores indicating poorer mental health.^27^ Scores of 0–7 are considered as non-indicative of anxiety or depression, while scores of 8–10 present mild cases, 11–14 present moderate cases and 15–21 present severe cases. Well-being was measured using the Warwick-Edinburgh Mental Well-being Scale short form, which encompasses both hedonic well-being (which focuses on experienced pleasure) and eudemonic well-being (which focuses on meaning and self-realisation).^28^ The scale is scored from 7 to 35 with higher scores representing higher levels of well-being. As recommended in validations, the raw scores were logit transformed prior to analysis.^29^ The New Economics Foundation suggests five levels of well-being based on quintile analyses of data in the UK Understanding Society Survey, 2009: poor (<22), below average (22–24), average (25–26), good (27–28) and excellent (>28).^29 30 30–35^

Our secondary outcome measures were self-efficacy and self-esteem. Self-efficacy was measured using the General Self-Efficacy Scale short form^31^; a six-item scale scored from 6 to 24 with higher scores indicating higher levels of self-efficacy. Self-esteem was measured using the single-item scale, which has been validated as an alternative to the Rosenberg Self-Esteem Scale.^32^ The single-item measure captures global self-esteem through asking participants if the statement ‘I have high self-esteem’ is ‘not very true of me (1)’ to ‘very true of me (5)’. This scale has been used in a number of intervention and longitudinal studies.^33–35^

In addition to these measures, we collected baseline data, a range of further variables. For demographic variables, we used self-report measurements of age, gender, ethnicity, income (<£16 000, £16 000–£30 000, £31 000–£60 000, £61 000–£90 000 and >£90 000) and employment status (unemployed, voluntary/temporary work, part-time work, full-time work and retired). We also asked whether participants were receiving psychological therapy, whether participants had any other health condition and how long ago they had been bereaved. In relation to musical engagement, we asked all participants whether they had previously sung in a choir, how confident they felt about singing (from 1 (not at all) to 5 (very)), whether they had attended musical concerts or performances in the past year and whether they had taken part in any other music activity in the past year.

### Statistics

The non-randomised design of this study means that the exchangeability of groups at baseline was not guaranteed. To compare potential baseline differences between the choir and control groups, we used one-way analyses of variance, χ^2^ test and Fisher’s exact test. To explore changes in mental health and well-being over time and between groups, we used linear mixed effects models (LMMs) with a random intercept and slope, and an unstructured covariance matrix of the random effects to allow for differences among participants both in their baseline mental health and change in mental health over time. Time was modelled first as a continuous variable to identify an overall time by group relationship (using a linear model as we found no evidence to support the use of a quadratic model), and then time was modelled as four separate time points to identify where specific changes occurred. Participants provided data with an average of 3.6 times across the four time points, providing 182 data points. Unlike some repeated measures analysis models that deal with missing data through list-wise deletion, LMMs make full use of the data set, so all 182 data points were included in the analysis. All models demonstrated normality of residuals and no evidence of heteroscedasticity.

We built-up our final model by considering nested models of relevant covariates. Model 1 was unadjusted, model 2 adjusted for demographic variables, model 3 additionally adjusted for health-related variables, model 4 additionally adjusted for musical engagement and attitudes to singing and model 5 additionally adjusted for length of time since bereavement. Model 5 was theoretically most suitable given that time since bereavement may have a confounding effect both on mental health and also on willingness to engage in new activities. This model was also confirmed as having the best fit through inspection of the log-likelihood ratio, Akaike’s information criterion and Bayesian information criterion. We calculated margins of response from the fully adjusted model and created profile plots to illustrate the time by group interactions. To explore the relationship between self-esteem and self-efficacy and our primary outcome measures, we used Pearson correlations of change scores over the 24 weeks.

Two participants during the study started a new medication and three started psychotherapy. Sensitivity analyses removing these participants from analyses did not affect the significance of any of the results so they were included in analyses to maximise power. All analyses were conducted using the Stata V.14.

## Results

### Demographics

Choir and control participants were well-matched statistically at baseline on all measures of mental health and well-being. Participants in the choir had a higher average age than those in the control group, and (relatedly) a greater proportion of them were retired. However, notably, fewer of those who selected to join a choir had sung in a choir previously, and the groups were well-matched statistically on all other measures of musical engagement (table 1).

**Table 1:** Baseline demographics of participants

	Control (n=29)	Choir (n=29)	P value
Age, mean (SD), years	52 (13)	62 (10)	0.001*
Sex, % female	89.7%	86.2%	>0.99^‡^
Ethnicity, % White British	79.3%	86.2%	0.73^‡^
Income, %			0.15^‡^
<£16 000	10.7%	30.8%	
£16 000–£30 000	25.0%	34.6%	
£31 000–£60 000	42.9%	26.9%	
£61 000–£90 000	3.6%	3.9%	
>£91 000	17.9%	3.9%	
Employment status, %			<0.001^‡^
Unemployed	6.9%	6.9%	
Voluntary	10.3%	13.8%	
Part-time work	27.6%	24.1%	
Full-time work	48.3%	6.9%	
Retired	6.9%	48.3%	
Currently having therapy, %	13.8%	6.9%	0.67^‡^
Pre-existing health condition, %	58.6%	24.1%	0.01^†^
Previously sung in a choir, %	55.2%	27.6%	0.033^†^
Confident in singing, %			0.19^‡^
1—not at all	13.8%	31.0%	
2	34.5%	27.6%	
3	31.0%	37.9%	
4	17.2%	3.5%	
5—very	3.5%	0%	
Attended a concert or performance in the past year, %	69.0%	48.3%	0.11^†^
Took part in a music activity in the past year, %	27.6%	17.2%	0.35^†^
Length of time since bereavement, %			0.73^‡^
0–6 months	20.7%	17.2%	
7–12 months	20.7%	34.5%	
1–2 years	13.8%	13.8%	
2–3 years	34.5%	31.0%	
3–5 years	10.3%	3.5%	
Depression, mean (SD)	4.83 (4.47)	4.83 (2.85)	>0.99*
Anxiety, mean (SD)	8.76 (4.82)	7.79 (3.92)	0.41*
Well-being, mean (SD)	22.81 (4.79)	22.46 (3.93)	0.76*
Self-efficacy, mean (SD)	18.14 (3.78)	17.69 (2.58)	0.60*
Self-esteem, mean (SD)	3.24 (1.21)	3.14 (0.83)	0.71*

*One-way analyses of variance.

†χ^2^ test.

‡Fisher’s exact test.

### Primary outcome measures

#### Symptoms of depression

**Figure 2:**
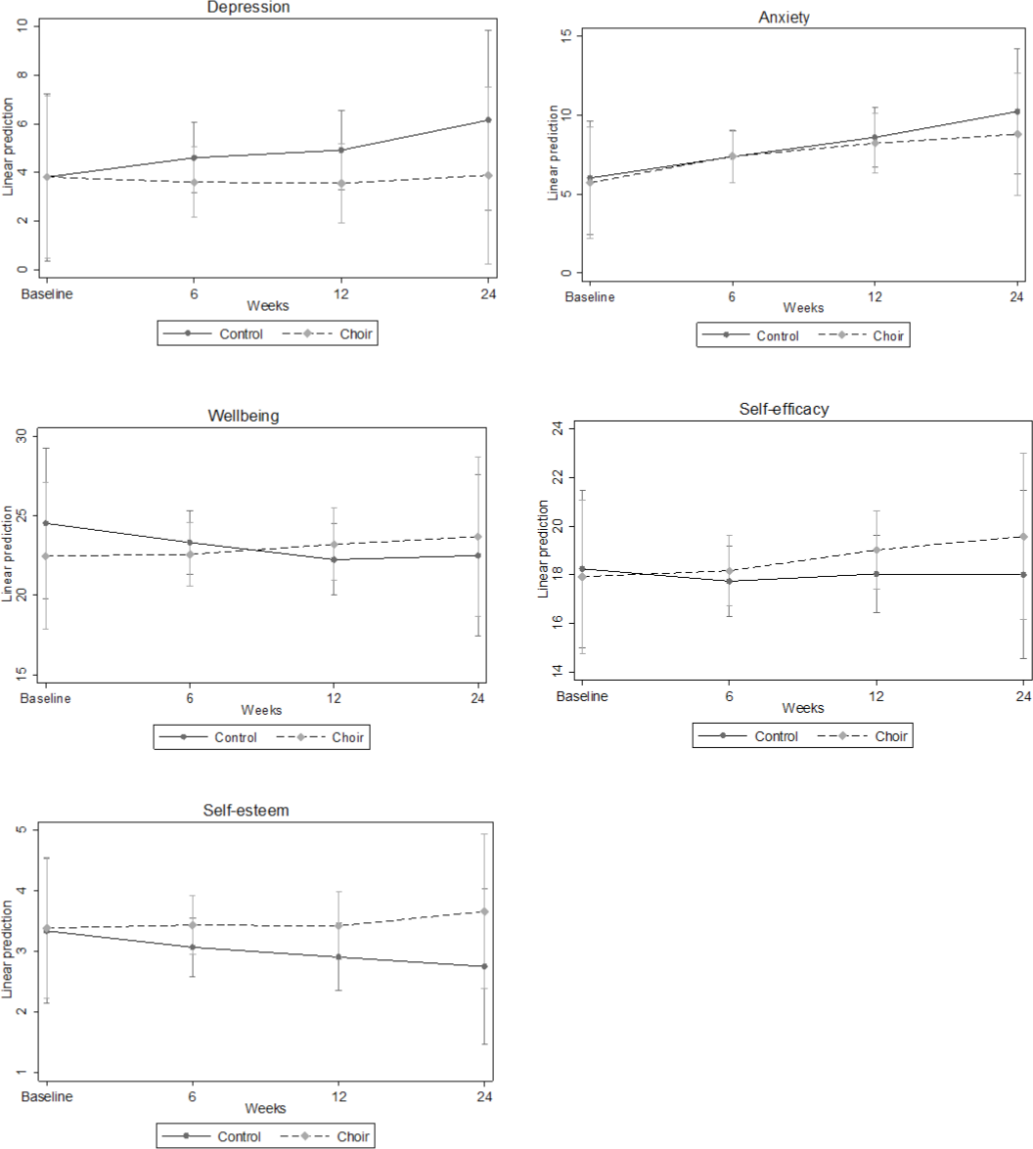
Predictive margins of time by group interaction with 95% CIs for people who had been bereaved in the past 5 years.

The LMM showed that there was a significant time by group interaction for symptoms of depression, with participants in the control group showing an increase in depressive symptoms while participants in the choir group showed a constancy across the 24 weeks (B=−0.74, SE=0.33, p=0.025). This difference was not apparent in the first 6 weeks (B=−1.02, SE=0.63, p=0.11), but was starting to show by week 12 (B=−1.38, SE=0.78, p=0.078) and was present by week 24 (B=−2.27, SE=1.01, p=0.024) (see figure 2).

#### Symptoms of anxiety

There was no significant time by group interaction for symptoms of anxiety (B=−0.36, SE=0.35, p=0.3). This difference was not apparent in the first 6 weeks (B=0.34, SE=0.72, p=0.64), nor by week 12 (B=−0.08, SE=0.85, p=0.93) and nor by week 24 (B=−1.15, SE=1.06, p=0.28), although there was a graphic demonstration of some tapering of increases in symptoms of anxiety in the choir group between weeks 12 and 24 (see figure 2).

#### Well-being

There was a significant time by group interaction for well-being, with participants in the control group showing a decline in well-being but participants in the choir group showing constancy across the 24 weeks (B=1.14, SE=0.46, p=0.013). This difference was not apparent in the first 6 weeks (B=1.33, SE=0.97, p=0.17) but was present by week 12 (B=3.02, SE=1.13, p=0.007) and was still present by week 24 (B=3.26, SE=1.40, p=0.02) (see figure 2).

### Secondary outcome measures

#### Self-efficacy

There was a significant time by group interaction for self-efficacy, with participants in the choir group showing a significantly greater increase in self-efficacy than participants in the control group (B=0.62, SE=0.31, p=0.044). This difference was not apparent in the first 6 weeks (B=0.76, SE=0.65, p=0.25) but was starting to show by week 12 (B=1.31, SE=0.76, p=0.086) and was present at week 24 (B=1.90, SE=0.94, p=0.044) (see figure 2).

#### Self-esteem

There was a significant time by group interaction for self-esteem, with participants in the control group showing a gradual decline in self-esteem but participants in the choir group showing constancy (with a marginal increase) in self-esteem (B=0.27, SE=0.12, p=0.021). This difference was not apparent in the first 6 weeks (B=0.32, SE=0.25, p=0.20) and not clear either by week 12 (B=0.47, SE=0.29, p=0.11) but was apparent by week 24 (B=0.87, SE=0.36, p=0.015) (see figure 2).

### Relationship between self-esteem and self-efficacy and primary outcome measures

Changes in both self-esteem and self-efficacy were associated with all measures of mental health and well-being. Specifically, improvements in self-efficacy and self-esteem were both associated with reductions in anxiety and depression and improvements in well-being (see table 2).

**Table 2:** Pearson correlations between changes in outcome measures over 24 weeks

	Symptoms of depression	Symptoms of anxiety	Well-being
Self-efficacy	r=−0.57, p<0.001	r=−0.34, p=0.015	r=0.53, p<0.001
Self-esteem	r=−0.46, p<0.001	r=−0.30, p=0.033	r=0.49, p<0.001

## Discussion

This study explored the effects of singing in a choir on mental health and well-being in people who have been bereaved. People who sang in a choir on a weekly basis had more stable symptoms of depression and levels of well-being as well as gradual improvements in their sense of self-efficacy and self-esteem. In contrast, those who did not sing on a weekly basis showed gradual increases in depressive symptoms, reductions in levels of well-being and self-esteem, and no improvement in their sense of self-efficacy. These results were independent of demographic covariates, health-related covariates, previous or current engagement in musical activities, attitudes to singing and length of time since bereavements.

This study supports and extends previous research suggesting that group singing can support people affected by cancer more broadly. Previous studies have found improvements in anxiety, depression and perceived health following 3 and 6 months of singing in people affected by cancer (including those who have been bereaved).^36^ Qualitative studies have highlighted the role of choirs in enhancing confidence and self-esteem in people affected by cancer,^37^ which echo the findings here. While our results did not find improvements in mental health, it did find greater stability in mental health across the 24 weeks among participants involved in the choir and, notably, participants in the choir did not experience decreases in mental health, as was seen in the control group. These findings also build on the few preliminary studies that have looked at singing in relation to bereavement. However, unlike some previous music therapy studies that have used singing as a way of delivering therapy or counselling,^38^ this intervention was not delivered by therapists as therapy, but instead constituted a mutual support intervention.^16^

In considering how singing led to changes in mental health, both self-esteem and self-efficacy appear to be key factors, with improvements in these two outcomes associated with the maintenance of stable mental health and well-being across the 24 weeks. This links with previous literature on music interventions for mental health that have found similar relationships between self-efficacy, self-esteem and mental health. For example, group drumming programmes have been found to enhance self-awareness, positive identity, self-prospection agency and control as well as reduce depression and anxiety and improve well-being.^39 40^ The learning opportunities provided by the musical engagement have emerged as a central mechanism behind these effects, both in studies of drumming and singing.^41 42^ A recent separate qualitative study of the choirs, involved in this study, also highlighted the role of learning in building resilience, including through supporting the development of confidence and coping skills,^43^ which could have supported the enhancement of self-efficacy and self-esteem found in these analyses. And several studies have highlighted how singing can improve self-confidence.^44^

It is also possible that the environment of a choir provides important elements to support mental health and well-being in participants. For example, Calhoun *et al* 11 propose that support for people experiencing grief should involve humility and respect not platitudes, tolerance of the non-rational, courage to hear, constancy and appreciation of paradox. The choir setting (which involved people affected in some way by cancer) ensured that the fellow choir members were familiar with the emotional challenges being experienced by those who had been bereaved and in a position to offer genuine empathy rather than platitudes, tolerance and understanding. The regularity of the weekly choir sessions provided constancy. And the songs sung (which involved a mixture of ballads and upbeat pop songs; some themed around loss and others unrelated) directly supported participants in engaging in paradoxes. Indeed, a recent qualitative study conducted by the authors of this paper found that the choirs identified the varied repertoire as one of the important features in building resilience among participants.^43^ Consequently, this combination of the mutual support provided by choirs, their support of learning and the environment they foster could explain the results found in this study.

This study had a number of strengths. We focused on a specific type of bereavement (bereavement from cancer) in order to differentiate from other types of bereavement, such as bereavement from sudden violent loss, which has been shown to affect mental health differently.^25^ The intervention at its core uses a well-researched model for providing support specifically for people affected by cancer that has been demonstrated to improve mental health in previous studies.^36^ We also tracked participants across a 6-month period of involvement. While the study was not randomised, our groups were statistically comparable in baseline mental health. Further, our use of LMMs using both random intercept and slope meant that we were able to model the effect not just of group but also of all of our covariates both on baseline mental health and change in mental health across the 24 weeks. However, it is still to be noted that our groups were not entirely comparable at baseline, with significant differences in age, employment status, previous choir experience and pre-existing health conditions. While we adjusted for these baseline levels and our results were found independent of all identified confounders, we cannot assume full exchangeability of the two groups. As a result, it is possible that confounders affected the exchangeability of our groups at baseline. In particular, while mental health, self-efficacy and self-esteem did not differ at baseline between groups, the fact that some people chose to take part in the choirs while others did not could have meant those in the singing group were more predisposed to benefit. Potential unmeasured confounders that could have influenced the decision to participate in the choirs (and also to experience improvements in mental health) include personality (although it is of note that a large observational study found no confounding effects of open personality types on cultural engagement and depression^45^) and factors relating to self-rated physical health and fatigue (especially given there were baseline differences in pre-existing physical health conditions). But it is worth noting that those who chose to join the choir had comparable levels of engagement with musical activities as those in the control group, and in fact had less previous experience of singing, suggesting that our experimental group was not biassed towards being more artistically engaged at baseline. Among other limitations, studies of adaptation following bereavement suggest that individuals tend to follow discrete trajectories in mental health, ranging from resilience without depression, to chronic grief with depression following loss, to persistent chronic depression before and after loss, and high preloss depression following loss that improves.^46^ It remains unknown whether singing works better for people following particular trajectories or whether it is an intervention capable of benefitting people on different trajectories. Of note is that levels of depression and below-average well-being in our sample at baseline were low, suggesting that our broad population in this study may have been following trajectories already associated with resilience. Nevertheless, it is still notable within this that the singing group demonstrated significant improvements compared with the control group. Finally, this study focused on a specific choir programme run by a specific charity. It remains to be clarified whether similar benefits are found from other choir or singing activities run by different organisations. Future studies could focus specifically on participants with diagnosed depression or anxiety to explore whether singing can be of particular support not just in subclinical symptoms but in recovery from mental illness.

In conclusion, weekly singing in a choir was found to support stability in mental health and well-being as well as increasing self-efficacy and self-esteem in people who had been bereaved by cancer. The particular environment and engagement provided by choirs suggest that they could be a promising mutual support intervention for people experiencing grief.

## Data Availability

Data are available on reasonable request.
